# Interactions among Collective Spectators Facilitate Eyeblink Synchronization

**DOI:** 10.1371/journal.pone.0140774

**Published:** 2015-10-19

**Authors:** Ryota Nomura, Yingzong Liang, Takeshi Okada

**Affiliations:** 1 Department of Education, The University of Tokyo, 7-3-1, Hongo, Bunkyo ward, Tokyo, Japan; 2 Graduate School of Engineering, The University of Tokyo, 7-3-1, Hongo, Bunkyo ward, Tokyo, Japan; Lanzhou University of Technology, CHINA

## Abstract

Whereas the entrainment of movements and aspirations among audience members has been known as a basis of collective excitement in the theater, the role of the entrainment of cognitive processes among audience members is still unclear. In the current study, temporal patterns of the audience’s attention were observed using eyeblink responses. To determine the effect of interactions among audience members on cognitive entrainment, as well as its direction (attractive or repulsive), the eyeblink synchronization of the following two groups were compared: (1) the experimental condition, where the audience members (seven frequent viewers and seven first-time viewers) viewed live performances *in situ*, and (2) the control condition, where the audience members (15 frequent viewers and 15 first-time viewers) viewed videotaped performances in individual experimental settings (results reported in previous study.) The results of this study demonstrated that the mean values of a measure of asynchrony (i.e., *D* ^interval^) were much lower for the experimental condition than for the control condition. Frequent viewers had a moderate attractive effect that increased as the story progressed, while a strong attractive effect was observed throughout the story for first-time viewers. The attractive effect of interactions among a group of spectators was discussed from the viewpoint of cognitive and somatic entrainment in live performances.

## Introduction

What makes a live performance so exciting? Multiple interactions within a group of spectators are likely a dominant factor. In recent years, many researchers have reported the entrainment of human behaviors such as body movement [1] or respiratory rhythms [2] during interpersonal communications. Behaviors of participants lead to particular patterns due to the repetitive interactions among participants. This somatic entrainment or synchronization is known as a basis of the shared group affect [3]. Although previous studies have paid little attention to the cognitive aspect of entrainment in live performances, some have suggested that spectators share a similar understanding of the meaning of the performance. This shared understanding can influence affinity or temporal cohesion, leading to enthusiasm and exhilaration by audience members during live performances. In actual theaters, however, it remains unclear how interactions among audience members affect cognitive entrainment. The purpose of the current study is to explore the effect of interactions among audience members in live performances on cognitive entrainment.

Some researchers have utilized eyeblink synchronization [4–6] as a possible objective measure of audience members’ cognitive synchronization during the observation of a live performance. It is possible that eyeblinks are associated with an individual’s cognitive process [7]. A study found that the cognitive load of a task increased rates of eyeblinks, even if it required no visual information [8]. Especially, occurrence timings of eyeblink loosely co-vary with basic cognitive processes such as the allocation and release of attention [4]. Hence, eyeblinks among audience members tend to occur at similar times when members are watching the same movie [4] or the same storytelling performance [5]. Moreover, the degree of synchronization (i.e., frequency of the co-occurrence of eyeblinks) depends on the viewing experience of audience members; frequent viewers have higher synchronization than first-time viewers do [6]. This finding implies that eyeblink occurrences seem to become more similar due to the domain knowledge mediated by influencing attentional process. If eyeblink synchronization in this sense is observed even when individuals are watching the same video separately [4], it is no wonder that this phenomena arises when individuals are collectively watching a live performance (i.e., *in situ*). In fact, an observational study has pointed out that eyeblinks co-occur more frequently than would be expected by chance in vaudeville settings ([5], Study 1). Thus, eyeblink synchronization could be used as a measure of cognitive entrainment among audience members in live performances.

If interactions among audience members have an attractive influence, it must facilitate eyeblink synchronization. In contrast, if the interactions among audience members have a repulsive effect, they must inhibit eyeblink synchronization. Given that domain knowledge tends to increase eyeblink synchronization [6], interactions would be expected to affect synchronization in different ways, depending on the viewing experience of the audience members. For instance, frequent viewers would be able to follow the course of a story using knowledge of the story, which would lead to increased synchronization. On the other hand, first-time viewers would try to use less cognitive resources to comprehend a story by relying instead on the information provided by other audience members (e.g., laughter and body movements), which would result in reinforced synchronization. To explore the effect of interactions on cognitive entrainment, as well as its direction (attractive or repulsive), this study compared the extent of eyeblink synchronization among spectators observed in two live performances (Orthodox version for frequent viewers and Modified version for first-time viewers) to that among the spectators in the control condition (reported in [6]), in which participants watched videos of the performances. If eyeblink synchronization occurred only due to the common inputs of the story-telling artist, then interactions would neither have attractive nor repulsive effects among audience members. In this case, the results would demonstrate no differences between these two groups (audience in situ vs. participants in the individual experimental setting [6]). On the other hand, if interactions among audience members were also due to eyeblink synchronization within the audience, systematic differences would be found. Moreover, if the collective nature of audience members was cooperative, interactions would have an attractive effect. If the collective nature was competitive, interactions would be repulsive. Therefore, in the former case, the results would show that eyeblinks would synchronize more in situ than in the individual experimental setting. In contrast, for the latter case, the results would occur in the opposite way. Regarding the cooperative nature, it could be hypothesized that it occurs by facilitating cognitive entrainment, which decreases each audience members’ cognitive cost. This hypothesis would be supported if the attractive effect were stronger for first-time viewers than for frequent viewers. However, if this cooperative nature was caused by mere somatic entrainment among audience members regardless of domain knowledge, there would be no difference found in eyeblink synchronization based on audience members’ viewing experience.

## Method

### Participants

Participants in the experimental condition (audience members) included 31 frequent viewers (mean age = 44.37, sd = 13.57) and 24 first-time viewers (mean age = 28.06, sd = 13.91) for two separate performances, respectively. We defined frequent viewers in this study as the participants who had viewed Rakugo (Japanese traditional storytelling performance) more than 10 times regardless of type. The reason why we adopted this criterion is that the mean number of viewing times in the daily lives of most Japanese people is usually three or four. This means that a participant who meets our criteria as a frequent viewer would seek opportunities to view the Rakugo performance more than the average Japanese person would. Seven (five male and two female) frequent viewers and seven (five male and two female) first-time viewers were selected from each audience group as the targets whose eyeblink responses would be observed. This is due to the limitation of the observation method: the experimenter could only detect the eyeblink responses of these participants by the recorded faces. As all of the observed targets sat facing forward instead of facing the audience, the targets were limited to obtaining visual information only from the performer. In other words, the participants were not able to look at each other’s faces or see each other’s eyeblinks. The participants in the control condition (see [6]) included 24 males and 36 females. Half of the participants were frequent viewers and the others were first-time viewers. The experimenter used the same criteria based on the participants’ viewing experience to divide them into frequent viewers and first-time viewers. All participants provided their written informed consent to participate in this study. This experiment was approved by Life Science Research Ethics and Safety, the Ethics Committee of the University of Tokyo.

### The storytelling artist and performed story

Rakugo is a traditional Japanese art of storytelling in which one artist plays many characters by changing face directions and sitting postures. Moreover, since the artist acts without lighting effects, visual information of the performer stays the same even when the scene changes. Therefore, the changes of characters and of scenes are interpreted by acting conventions among the performer and audience members. In order to understand the story, the audience members themselves have to work to make non-obvious segmentations on the continuous performance. In order to investigate the particular impact of story-telling performances, the authors used Rakugo performances, on which only minimal visual and sound effects are added, for this experiment. Because each artist is classified into three grades in accordance with their acting abilities, the author was able to validate the quality of the artist in this study. The artist of the live Rakugo performance was Kokontei Bungiku (34-year-old with 10 years’ worth of experience as a performer) who is one of the highest-graded artists. The performed story was one of the classic Rakugo repertories, “Nibansenji,” which literally means the second brew of tea or decoction. The performance for frequent viewers was conducted in the style of traditional vaudeville storytelling performances in everyday theater (Orthodox version). The performance for first-time viewers was modified to allow the first-time viewers to better comprehend the content of the story (Modified version). The total time of the performances were 3022 seconds (s; 50 minutes (min) 22 s) and 3220 s (53 min 40 s), respectively. For the first-time viewers, the artist took a few minutes to explain the traditional way of viewing this type of storytelling performance. In the experiment settings, the experimenter used the video clips of the live performances.

### Data collection

**Fig 1:**
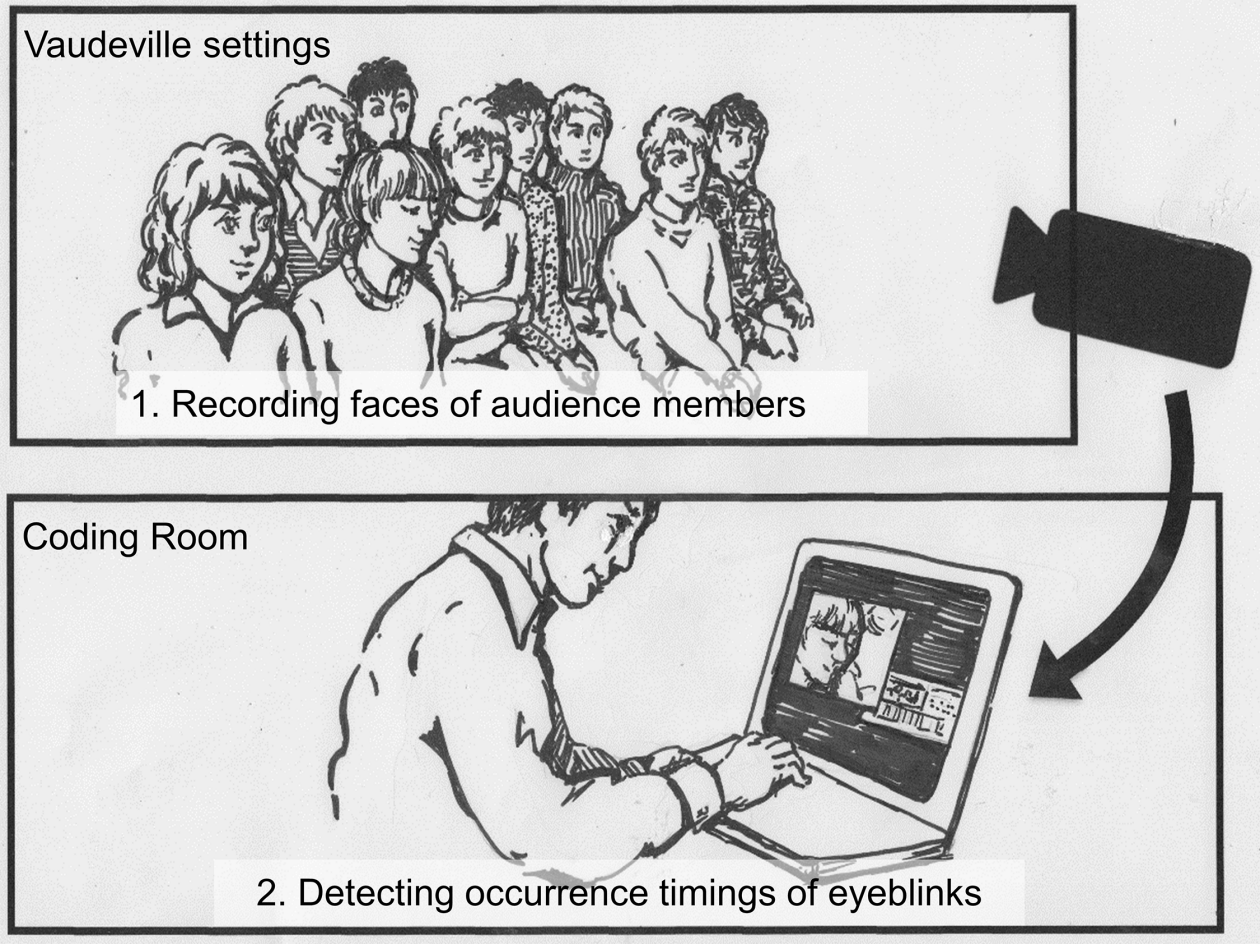
Illustration of an analyzer coding eyeblink occurrence timings.

To detect the eyeblink responses, the analyzer utilized ELAN 4.5.1 (Max Planck Institute for Psycholinguistics, Nijmegen), which was developed for discourse analysis. When using this software, an analyzer can easily record many types of annotations on utterances and gestures (e.g., the onsets or offsets of a particular gesture). The onset of an eyeblink was defined as the frame prior to the frame in which the pupils were covered by the eyelids after a target audience member started blinking. An analyzer who had two years’ worth of Rakugo-performing experience coded the eyeblink responses using the video recording of the audience (33.4 Hz) that was muted to eliminate the possibility of being influenced by the recorded voice of the performer (Fig 1). An analyzer also reduced the playing speed by approximately 60% of the original video, thus preventing the analyzer from missing the target’s eyeblinks due to his own eyeblinks. If the motion of eyelids did not cover the whole eye surface area, it was coded as a muscle artifact and was not used in the analysis. To confirm coding reliability, the first author separately coded one of the targets using the same procedure. As both coders concurrently identified more than 95% of the eyeblinks, the data (S1 File) coded by the analyzer were used for the consequent analysis.

### Procedure

Participants were invited to enter the vaudeville setting recreated in the laboratory room. Six seats (three seats by two lines) were reserved for the first six participants of the experiment. The other seats were free seating. For first-time viewers, the experimenter explained the procedure of Rakugo performances (e.g., a storytelling performer changes his voice and turns his face to play many characters by himself). Then, the artist went to the center of the stage while a classic Japanese theme song played on the speakers. The artist sat down on the square cushion to start the performance. After the performance, the artist left the room. The experimenter and assistants distributed questionnaires to the participants in order to obtain their demographic variables and assess their domain knowledge regarding this kind of performance.

### Distance-based analysis of blink (spike) trains: Asynchrony

**Fig 2:**
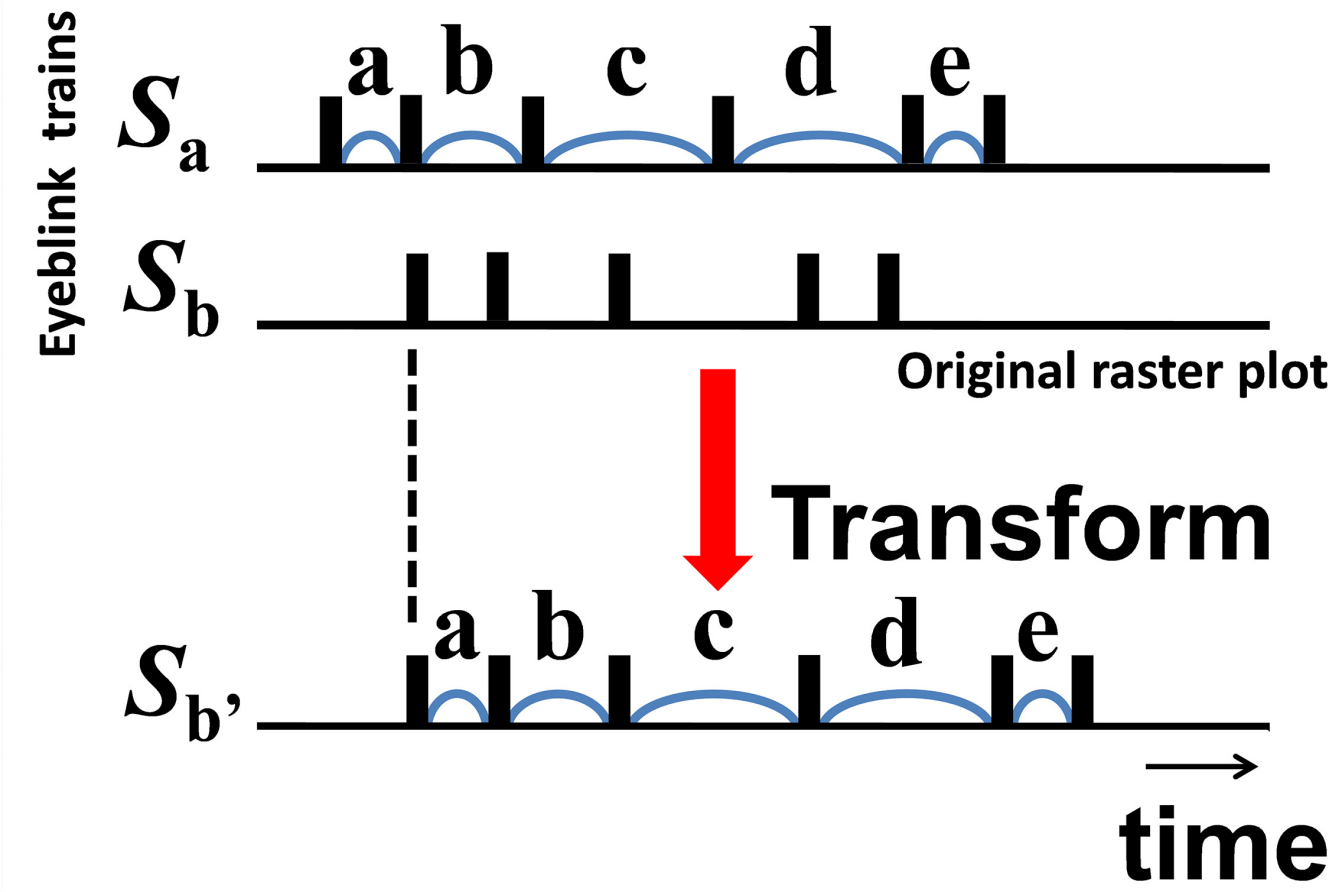
Schematic illustration of a method to evaluate costs to transform a train to another train to calculate D^interval^.

Victor and Purpura [9] proposed a method to quantify the asynchrony of two particular spike trains (e.g., the time series of intermittently firing neurons) focusing on the difference of spike timings, *D* ^interval^. This method does not assume a Euclidean notion of distance. Rather, it adopts a metric space to define the distance, and then the method can be applied to the time trains of eyeblink occurrence [6]. In this study, *D* ^interval^ was used to evaluate the distances of two different blinking trains (Fig 2). The distance between the two spike trains, *S*_a_ and *S*_b_, is equal to seeking a path of the minimum cost, which transforms *S*_a_–*S*_b_, with IBIs (a, b, c, d, e) equal to *S*_b_’. If the original pattern of *S*_b_ is more similar to *S*_a_, the cost of transforming is lower. Hence, D^interval^ quantifies the asynchrony between two particular time trains. In this study, the analysis unit was set to 250 milliseconds (ms) to maintain a format of results similar to that of the control condition results (reported by [6]). In other words, the entire video recording was divided into many time windows of 250 ms width (i.e., bins). To evaluate the asynchrony of each scene during the performance, time trains of 5 min of performance time each (i.e., 1200 bins = 4 bins/s × 60 s × 5 min) were used for calculations as in the control condition. Welch’s tests of the mean D^interval^ *in situ* vs. the mean D^interval^ in the experiment were performed for each scene using Bonferroni-adjusted *p*-values.

## Results

**Fig 3:**
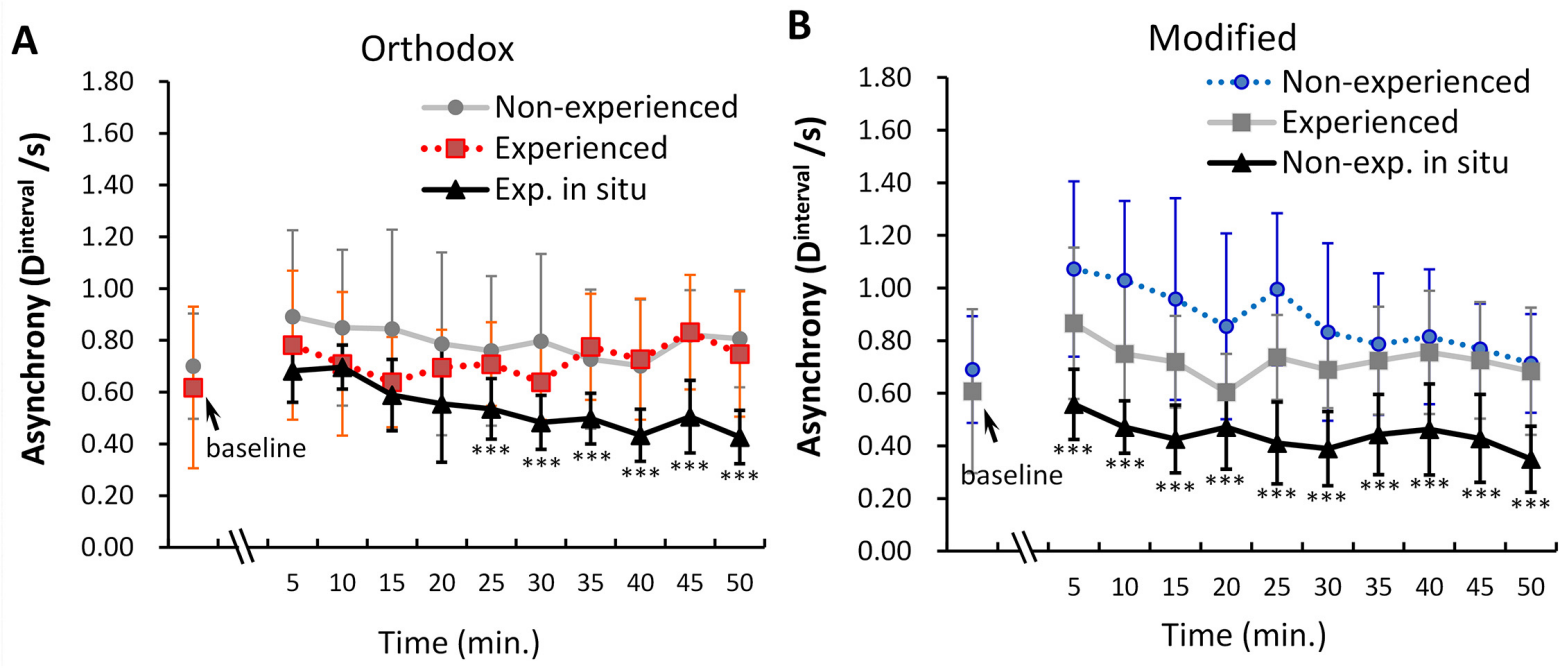
Asynchrony of eyeblinks among participants at each scene (5 min) during observation of the performance. **(A)** Orthodox live (black line) and videotaped (orange dashed line) performances for frequent viewers, and **(B)** modified live (black line) and videotaped (blue dashed line) performances for first-time viewers. Both dashed lines and gray lines show data reported by [6]. Error bars show the sd. Asterisks indicate the *p*-values of Welch’s tests, which were performed for each scene between the mean D^interval^ *in situ* vs. the mean D^interval^ in the experiment. Bonferroni-adjusted *p*-values were used. ***p < .001.

Nomura et al. [6] reported that the eyeblinks of frequent viewers synchronized than that of first-time viewers during the first 30 min of the performance in both videos (Orthodox version and Modified version). This effect of viewing experience on eyeblink synchronization was especially stronger in the Modified version (Fig 3B). In contrast, the results observed in the live performances in this study demonstrated that both the *D* ^interval^s of frequent viewers and that of the first-time viewers were much lower than those of each control condition (black lines in Fig 3A and 3B), indicating that eyeblinks synchronized *in situ* rather than in the individual control condition, regardless of the version of performances. For frequent viewers, there was no difference in the asynchrony of eyeblinks at the starting point of the story despite the format of the Orthodox version of the performance (Fig 3A, orange line and black line). However, the asynchrony (*D* ^interval^s) of the audience members’ eyeblinks *in situ* decreased as the story progressed (from the fifth scene to the tenth scene, respectively: *t* (37.10) = 5.77, *p* < .001; *t* (37.10) = 5.80, *p* < .001; *t* (61.19) = 9.45, *p* < .001; *t* (70.70) = 9.26, *p* < .001; *t* (43.02) = 8.75, *p* < .001; *t* (71.43) = 9.84, *p* < .001). As a result, asynchrony was lowest during the last 5 min of the performance, including the final remark (punch line). In contrast, in the performance of the Modified version (Fig 3B, blue line and black line), throughout the whole story, there were major gaps between the asynchrony (*D* ^interval^s) gained from *in situ* and that acquired from the experiment for first-time viewers (from the first scene to the tenth scene, respectively: *t* (118.81) = 8.61, *p* < .001; *t* (118.81) = 10.06, *p* < .001; *t* (120.32) = 8.97, *p* < .001; *t* (98.26) = 6.50, *p* < .001; *t* (70.62) = 11.91, *p* < .001; *t* (99.82) = 8.42, *p* < .001; *t* (72.98) = 7.08, *p* < .001; *t* (57.20) = 6.97, *p* < .001; *t* (57.43) = 6.93, *p* < .001; *t* (100.44) = 7.79). The asynchrony of the first-time viewers’ eyeblinks *in situ* was lower (Fig 3A, black line) even when compared to that of the eyeblinks of frequent viewers (Fig 3B, black line).

## Discussion

### Comparison between frequent viewers and first-time viewers

The purpose of the current study is to explore the effect of interactions among audience members on the cognitive aspect of entrainment in live performances. To assess the effect of interactions, the eyeblink synchronization of audience members during live performances was compared to that of audience members in individual experiments. The results demonstrated that interactions among audience members facilitate eyeblink synchronization. Although this attractive effect was found in both the Orthodox version for frequent viewers and the Modified version for first-time viewers, the estimated effect of interactions for first-time viewers was stronger than that of frequent viewers. First-time viewers would have to spare cognitive resources to comprehend a story. Hence, the cognitive load would be relatively higher for first-time viewers than frequent viewers. Previous research [7, 8] reported that as the cognitive load increased, eyeblink rate increased. In this study, however, first-time viewers synchronized more their eyeblinks (i.e., showed lower asynchrony, *D* ^interval^, as compared to the experienced viewers). Occurrence timings of eyeblinks were found to be related to change in attentional state regardless of stimulus modality [10]. If one audience member can adopt clues of attentional processing from other audience members in a collective situation, even first-time viewers would lead cognitive entrainment at a smaller cognitive cost. It is suggested that first-time viewers enjoy the story by unintentionally utilizing other audience members’ responses as clues for cognitive processing.

On the other hand, frequent viewers appeared to be relatively less influenced by the temporal responses of other audience members, since they internalized viewpoints of this domain based on their many experiences. An existing study [6] reported that frequent viewers synchronized their eyeblinks by watching the same story-telling performance. Thus, frequent viewers seemed to enjoy the story based on individual cognitive processing and using their own domain knowledge.

Owing to the fact that all target audience members faced forward in their audience seats, they seemed to be influenced mainly by the laughter of other audience members instead of by visual information. A theory of humor [11] has suggested that further elaboration increases the experience of subjective humor. Laughter could be one of the clues indicating the occurrence of elaboration [12].

### Advantages of the current study and future directions

This study analyzed eyeblink synchronization in order to shed light on the cognitive entrainment that emerges because of interpersonal communications. This approach could provide a new perspective from which the dynamics of collective human behaviors in a temporally shared field might be examined. Audience members appeared to attract each other, leading to a mutual entrainment [13]. Eyeblink synchronization was also observed when individuals were watching the same storytelling performance separately [5], during which a forced entrainment [13] between the performer and each audience member would occur. In the actual theater, these two entrainments would occur in complex ways. From the viewpoint of a complex system, eyeblink synchronization is hypothesized to be a synchronization of multiple agents’ periodic behaviors induced by the common inputs [14]. This performer-audience system includes both top-down inputs and bottom-up emergent processes [15]. Attractive effects of interactions among audience members suggest that partly depending on other members who have received the same inputs could possibly be a mechanism of self-adaption [16] based on visual cues that are used in collective viewing situations. From this perspective, becoming an expert narrative artist is a process that involves acquiring adaptive control strategies to be used in uncertain situations where unexpected patterns of audiences’ response usually occur.

However, in the current study, the interactions among audience members were estimated as the total mass. Thus, the results only roughly illustrate a sketch of the time developments of cognitive entrainment. In future research, it would be necessary to develop a model of an entrainment system between a performer and the spectators. Somatic entrainment and cognitive entrainment within an audience are still not well understood. Does somatic entrainment lead to cognitive entrainment, does cognitive entrainment lead to somatic entrainment, or is it mutual? This is a key question, as situations in which a speaker performs in front of spectators are ubiquitous in human cultures. The elaborated model must provide universal findings and strategies that are applicable to other kinds of oral performance, such as speeches, presentations, lectures, and so on.

## Supporting Information

S1 File Supporting Information Data. This file contains data including condition, participants’ ID, the blink numbers, time stamp, and time stamp (second).
(CSV)
